# Relationship of Gallbladder Diseases with Sociodemographic Characteristics, Lifestyle, and Chronic Diseases in Northeastern China

**DOI:** 10.3390/ijerph15112596

**Published:** 2018-11-21

**Authors:** Qi Kang, Guojun Kang, Rixin Li, Xiaojing Zhu, Yaqin Yu, Qiong Yu

**Affiliations:** Department of Epidemiology and Biostatistics, School of Public Health, Jilin University, Changchun 130021, China; kangqi17@mails.jlu.edu.cn (Q.K.); kanggj17@mails.jlu.edu.cn (G.K.); rxli17@mails.jlu.edu.cn (R.L.); zhuxj17@mails.jlu.edu.cn (X.Z.); yuyaqin5540@163.com (Y.Y.)

**Keywords:** gallbladder disease, relationship, cross-sectional study

## Abstract

Background: Gallbladder diseases are common in Jilin, China. However, there have been few previous studies on this disease. Our study used the chronic disease database in Jilin Province to study the factors correlated with gallbladder diseases. Methods: A total of 21,435 people were selected from the Jilin Province adult chronic disease survey conducted in 2012. Multistage stratified random cluster sampling was used in this cross-sectional study. Multiple logistic regression analysis was used to explore the independent associations of different factors with gallbladder diseases. Results: There were 1876 people with gallbladder diseases, and the prevalence of the diseases was 8.8% (males 4.4%, females 12.8%). Multivariate logistic regression analysis showed that female (prevalence odds ratio (POR) = 3.13, 95% confidence intervals (CIs): 2.76–3.55), older people (30–45 years (POR = 2.79, 95% CIs: 2.06–3.77), 45–60 years (POR = 4.26, 95% CIs: 3.17–5.73), 60–79 years (POR = 4.72, 95% CIs: 3.48–6.41)), people living in rural areas (POR = 1.65, 95% CIs: 1.49–1.82), smoking (current smoker (POR = 1.15, 95% CIs: 1.01–1.31), former smoker (POR = 1.37, 95% CIs: 1.13–1.66)), high frequency of eating seafood (POR = 0.77, 95% CIs: 0.63–0.93), and high frequency of eating soy products (POR = 0.50, 95% CIs: 0.44–0.58) were associated with gallbladder diseases. Conclusions: We found that there were some factors associated with gallbladder disease, and there needs to be further studies to confirm these associations.

## 1. Introduction

With the development of society, our concern about diseases has shifted from the traditional focus on diseases such as cancer to the prevalence of chronic diseases. Indeed, chronic diseases pose a serious threat to human health [1]. Gallbladder disease is a common chronic disease, and its prevalence varies from 4% to 73% in different regions [2], bringing serious financial burden to regional governments. Gallbladder diseases are common in the population, especially in some European countries and the United States [3,4]. However, there have been few reports regarding the prevalence of gallbladder diseases in Asian countries.

Most of the studies on the related factors of gallbladder diseases have been conducted in western developed countries. Previous studies from the United States have suggested that smoking (both past and current) as well as saturated fat and cholesterol intakes are important risk factors. On the other hand, fruits, vegetables, and foods rich in dietary fiber act as protective factors against gallbladder diseases [5]. Another study from the United States confirmed that the protein intake is associated with lower risk of gallbladder diseases [6]. The results of the above studies show that diet and lifestyle habits may be associated with gallbladder diseases. In China, the largest developing country in the world, people’s living standards have improved significantly in the past 10 years, and the health problems derived from lifestyle changes have attracted much attention. In particular, the incidence of gallbladder diseases has increased year by year. Therefore, in this study, we conduct a study to investigate the prevalence of gallbladder diseases in the northeastern Chinese population and the association of diet and lifestyle with gallbladder diseases. There have been some earlier studies showing that diabetes and dyslipidemia are related to the occurrence of gallbladder diseases [7,8,9]. Therefore, we also considered the relationship between several other common chronic diseases and gallbladder diseases in this study.

At present, there is no nationwide survey data on gallbladder diseases in China. Through the investigation of chronic diseases in Jilin Province, we aimed to determine the prevalence of gallbladder diseases in China and find possible association factors for gallbladder disease.

## 2. Materials and Methods

### 2.1. Study Population

This study was part of a cross-sectional survey of chronic diseases in Jilin Province conducted from June 2012 to August 2012 [10,11]. The resident population of Jilin Province was 27.5 million at the time. In order to ensure the representativeness of the survey samples, the differences in economic level, the uniformity of geographic coverage, and the feasibility of the survey were considered in different regions of the province. A multistage stratified random cluster sampling method was adopted. (For more information on crowd sampling, please refer to the articles previously published by our group [12].) According to the sampling plan, a total of 32 districts/counties in nine cities of the province were taken as investigation points. We considered stratification factors, such as urban and rural areas, gender, and age groups, and sampled them according to one thousandth of the province’s total population. The number of planned survey samples was 23,050. However, due to the weather, the determined sample size was 21,449. In the end, 21,435 people completed the investigation. The response rate of subjects in this study was 93%.

Our study was conducted in accordance with the bioethics principles of the Declaration of Helsinki. The study was authorized by the Ethics Committee of Jilin University School of Public Health (Reference Number: 2012-R-011) and the Bureau of Public Health of Jilin Province (Reference Number: 2012-10). All participants voluntarily joined this study with informed consent.

### 2.2. Data Collection

Survey methods were divided into centralized investigation and household survey. The staff at the investigation points made an appointment according to the sample list. The investigators conducted one-on-one surveys with the research subjects. Then, a physical examination, blood glucose test, and blood sample collection were taken of the survey subjects. Within one week after the investigation ended, the investigation team fed back the results and the reports of the lipids test to the investigation points and informed respondents by telephone to collect them. Investigators participated in systematic training and conducted a preinvestigation before the formal investigation. All instruments were corrected before investigation. The survey was verified on site and afterwards to ensure its correctness. We used the parallel double input method to record the data.

The gallbladder diseases we studied included cholecystitis and gallstones. All patients with gallbladder diseases in the past year had been diagnosed by doctors in hospitals above the county level. Doctors diagnosed gallbladder diseases based on cholecystitis and gallstones according to the International Classification of Diseases, 10th Revision (ICD-10) definition. Annual prevalence was used to indicate the distribution of gallbladder disease in the past year. The sociodemographic factors and lifestyle of the respondents were collected by the investigators. People who “never drink” meant they have drunk no more than 12 glasses of alcohol throughout their life. “Never smokers” meant they have never smoked in their life or their total number of smokes did not exceed 100 cigarettes. “Former smokers” meant people who have smoked more than 100 cigarettes in their life but do not smoke now. Current smokers meant they have smoked more than 100 cigarettes in their life and still smoke [13].

According to the living standards of residents in Jilin Province, we divided the monthly income per capita of the family into three levels: low (<1000 yuan/month), middle (1000–3000 yuan/month), and high income (≥3000 yuan/month). Regular diet referred to eating three meals a day on time. Taste of diet included salty, light, and suitable, and the subjects were allowed to choose their own preferences regarding their tastes. The consumption frequency of vegetables, fruits, meat products, seafood, soy products, and milk were divided into three categories: everyday, sometimes, and few or never. Sometimes eat referred to the intake of the abovementioned foods at a frequency of ≥2 times/week. Few or never eat referred to the frequency of ingestion of these foods being <2 times/week. The above criteria came from the recommendations of the local health bureau.

Hypertension was defined as systolic blood pressure (SBP) of ≥140 mmHg or diastolic blood pressure (DBP) of ≥90 mmHg. Both SBP and DBP were taken as the average of two measurements, which were conducted at least 5 min apart [14]. Hyperlipidemia was defined as total cholesterol (TC) of ≥5.2 mmol/L, triglyceride (TG) of ≥1.7 mmol/L, high-density lipoprotein cholesterol (HDL-C) of <1.0 mmol/L, or low-density lipoprotein cholesterol (LDL-C) of ≥3.4 mmol/L [15]. Diabetes was defined as a history of taking hypoglycemic agents and diabetic or fasting blood glucose of ≥7.0 mmol/L [16].

### 2.3. Statistical Analysis

Data was input in the computer by Epidata (Version 3.1, Odense, Denmark) and analyzed by SPSS (Version 24.0, IBM SPSS, IBM Corp, Armonk, NY, USA). Categorical variables were expressed as frequency. Comparisons between groups with and without gallbladder diseases with regard to sociodemographic, life behaviors, and common chronic diseases were conducted using Rao–Scott x^2^ test. All the variables that significantly differed between the two groups in the univariate analyses were included in Model 2 (multivariate logistic regression) by stepwise method to examine their independent associations with gallbladder disease. Prevalence odds ratio (POR) with 95% confidence intervals (CIs) was calculated in Model 1 (univariate logistic regression) and Model 2. *p* < 0.05 (two-sided) was considered as statistically significant.

## 3. Results

The study involved 21,435 subjects, of whom 10,337 were males and 11,098 were females. The gender ratio (males: females) was 1:1.07, and the average age was 47.07 ± 13.41 years (46.16 ± 13.95 years in males and 47.91 ± 12.82 years in females). The annual prevalence of gallbladder diseases was 8.8% (males 4.4%, females 12.8%).

Table 1 shows the relationship between sociodemographic characteristics, living behaviors, and common chronic diseases and gallbladder diseases according to the results of univariate analysis. Variables like gender, age, residence, education, family income, smoking habits, drinking, regular diet, taste of diet, eat breakfast every day, physical exercise, diabetes, hyperlipidemia, and hypertension were correlated with gallbladder diseases as was the intake frequency of fruit, meat, seafood, soy products and milk. In addition, we also analyzed the relationship between sleep time and gallbladder disease and found that sleep duration was also closely related to gallbladder diseases.

We included these significant factors in Model 2 by stepwise method. Our analyses revealed that female (POR = 3.13, 95% CIs: 2.76–3.55), older people (30–45 years (POR = 2.79, 95% CIs: 2.06–3.77), 45–60 years (POR = 4.26, 95% CIs: 3.17–5.73), 60–79 years (POR = 4.72, 95% CIs: 3.48–6.41)), people living in rural areas (POR = 1.65, 95% CIs: 1.49–1.82), smoking (current smoker (POR = 1.15, 95% CIs: 1.01–1.31), former smoker (POR = 1.37, 95% CIs: 1.13–1.66)), the consumption frequency of seafood (eat every day (POR = 0.77, 95% CIs: 0.63–0.93), sometimes eat (POR = 0.84, 95% CIs: 0.76–0.94)), and consumption frequency of soy products (eat every day (POR = 0.50, 95% CIs: 0.44–0.58), sometimes eat (POR = 0.56, 95% CIs: 0.48–0.65)) were associated with gallbladder diseases (Table 2).

## 4. Discussion

Gallbladder diseases seriously affect the quality of life of patients. In this cross-sectional study, we investigated the annual prevalence of gallbladder diseases and related factors. The annual prevalence of adult gallbladder diseases in China’s Jilin Province was 8.8% (4.4% for men and 12.8% for women). A survey based on America of gallbladder diseases also yielded similar results [17,18], but the results of an investigation based on the prevalence of gallbladder diseases in India were lower than ours [19]. However, the two studies were very different from our research. Firstly, Americans are diverse, and there have previously been reports that ethnicity is also an important factor in the association of gallbladder diseases. Secondly, the Indian study included gallstones and gallbladder cancer, i.e., the definition of gallbladder diseases was different from ours. Therefore, the above two studies do not provide much reference to us, and a study for the Chinese population was necessary. Our findings are mainly applicable to the population of northern China but have an important reference value for the study of gallbladder diseases in other parts of China.

In line with most studies, we found females had a higher correlation with gallbladder diseases than males [5,20]. Some researchers have found that the history of reproductive health, multiple births, and oral contraceptives increase the risk of gallbladder diseases [21,22]. The reports from these literatures show that some female hormones make them more susceptible to gallbladder diseases than males. The reason for this phenomenon may also be the existence of some special genes in the female body, making them more susceptible to gallbladder diseases than males.

Few scholars have compared the prevalence of gallbladder diseases between city and rural areas. In this study, we found that there was a greater correlation between people living in rural areas and gallbladder diseases compared to those living in city areas. The reason for this result may be that, despite of the level of hygiene and medicine, people living in rural areas have a single diet compared to city people. In rural China, as people’s living standards improve, more and more people are starting to eat more meat and regard this as a symbol of quality of life rather than paying attention to a reasonable mix of diet. It is well known that some specific diets usually have connection to certain diseases.

Elderly people were at higher risk of gallbladder diseases than young people. This conclusion was consistent with others studies [23,24]. A possible reason for the result may be the marked aging of the function of organs, meaning resistance to the disease among the elderly is limited. There is also a possibility that older people suffer from a variety of chronic diseases at the same time, and other diseases promote the occurrence of gallbladder diseases.

In good agreement with other studies, we found that smoking is associated with gallbladder diseases. However, its biological mechanism remains unclear [25,26]. Other studies have shown that smoking may affect serum lipid and lipoprotein concentrations, and these serological changes may be associated with gallbladder diseases [27]. In our study, we divided smoking habits into three categories: current smokers, former smokers, and never-smokers. We found that former smokers were 1.37 times more likely to have gallbladder diseases than those who had never smoked, and current smokers were 1.15 times more likely to have gallbladder diseases than those who had never smoked. The reason for these results may be that we did not count the duration of smoking in this survey. Therefore, there may have been cases where those who had quitted smoking had smoked for a longer period than those who are currently smoking. The mechanisms by which smoking is associated with gallbladder diseases are complex and require further study.

Our results suggested that the higher the frequency of intake of seafood and soy products, the lower was the likelihood of gallbladder diseases. Some studies have shown that vegetables protein intake is associated with lower risk of gallbladder diseases and that there is no association between animal protein intake and risk of gallbladder diseases [6,28]. Seafood contains large amounts of unsaturated fatty acids, most of which are long-chain polyunsaturated fatty acids, and can reduce the cholesterol saturation in the bile, meaning the ability of bile to dissolve cholesterol is always within the normal range [29]. At the same time, seafood and soy products contain less triglycerides and cholesterol, which are also conducive to maintaining the normal metabolism of bile [30]. All these are good for maintaining the normal function of bile. Therefore, we believe that regular intake of seafood and soy products may reduce the occurrence of gallbladder disease.

Some studies have investigated the association between sleep duration and chronic diseases. Among them, sleep duration and gallbladder disease has been shown to be related [31]. In our study, we also included sleep duration in the univariate analysis. However, we did not find a similar association in multivariate analysis. The reason for this variance may be because previous studies only included people in the 18–59 age group.

This research comprised a large sample size, and the sample was also sufficiently representative. However, the study still had some limitations. First, this study was a cross-sectional study with the primary purpose being to describe the annual prevalence of gallbladder disease and to find possible associated factors; therefore, it did not test or validate the cause hypothesis. Second, although this was a large epidemiological survey, there were still some meaningful variables that were not included in the questionnaire. Third, some variables such as smoking, drinking, and some dietary factors were subjectively reported by the subjects and were not the result of objective testing, meaning there may have been some bias. Finally, the results of this study can only represent the annual prevalence of gallbladder disease in the population of northern China. Therefore, the results should be cautiously used when explaining the annual prevalence of gallbladder disease in other parts of the country.

## 5. Conclusions

The results of our study suggest that demographic characteristics and dietary intake are associated with gallbladder disease. In particular, female and older people have significant correlations with gallbladder disease. At the same time, intake of seafood and soy products is also associated with gallbladder disease. A larger research is needed in the future to confirm these findings.

## Figures and Tables

**Table 1:** Sociodemographic characteristics, living behaviors, and common chronic diseases among subjects with and without gallbladder diseases (univariate analysis).

Variable	No Gallbladder Diseases n (%)	Gallbladder Diseases n (%)	x^2^	*p*
Female	9681 (49.50%)	1417 (75.50%)	464.77	<0.001
Age			307.20	<0.001
18–29	2466 (12.60%)	49 (2.60%)		
30–44	6112 (31.20%)	416 (22.20%)		
45–59	7375 (37.70%)	902 (48.10%)		
60–79	3606 (18.40%)	509 (27.10%)		
Residence (Rural)	9110 (46.60%)	1173 (62.50%)	174.47	<0.001
Education (year) >9	8512(43.50%)	562(30.00%)	128.98	<0.001
Family income (yuan)			160.25	<0.001
<1000	7289 (37.30%)	974 (51.90%)		
1000–3000	9477 (48.50%)	713 (38.00%)		
>3000	2018 (10.30%)	122 (6.50%)		
Other ^a^	775 (4.00%)	67 (3.60%)		
Smoking habits			42.41	<0.001
Never smoker	11738 (60.00%)	1254 (66.80%)		
Current smoker	6259 (32.00%)	464 (24.70%)		
Former smoker	1562 (8.00%)	158 (8.04%)		
Drinking	6484 (33.20%)	344 (18.30%)	173.06	<0.001
Regular diet	15748 (80.50%)	1542 (82.20%)	3.10	0.078
Taste of diet			7.94	0.019
Suitable	6817 (34.90%)	636 (33.90%)		
Salty	7437 (38.00%)	675 (36.00%)		
Light	5305 (27.10%)	565 (30.10%)		
Staple food (Rice)	16709 (85.40%)	1556 (82.90%)	8.40	0.004
Eat breakfast every day	16164 (82.60%)	1650 (88.00%)	34.39	<0.001
Fresh vegetables			6.40	0.041
Few or never eat	295 (1.50%)	32 (1.70%)		
Eat everyday	17482 (89.40%)	1705 (90.90%)		
Sometimes eat	1782 (9.10%)	139 (7.40%)		
Fruit			29.15	<0.001
Few or never eat	3753 (19.20%)	457 (24.40%)		
Eat everyday	10172 (52.00%)	906 (48.30%)		
Sometimes eat	5634 (28.80%)	513 (27.30%)		
Meat			203.63	<0.001
Few or never eat	4348 (22.20%)	677 (36.10%)		
Eat everyday	7003 (35.80%)	464 (24.70%)		
Sometimes eat	8208 (42.00%)	735 (39.20%)		
Seafood			105.73	<0.001
Few or never eat	8859 (45.30%)	1078 (57.50%)		
Eat everyday	2135 (10.90%)	134 (7.10%)		
Sometimes eat	8565 (43.80%)	664 (35.40%)		
Soy products			176.27	<0.001
Few or never eat	1656 (8.50%)	332 (17.70%)		
Eat everyday	12018 (61.40%)	1002 (53.40%)		
Sometimes eat	5885 (30.10%)	542 (28.90%)		
Milk			67.03	<0.001
Few or never eat	12045 (61.60%)	1335 (71.20%)		
Eat everyday	3214 (16.40%)	228 (12.20%)		
Sometimes eat	4300 (22.00%)	313 (16.70%)		
Physical exercise			40.40	<0.001
Often	5749 (29.40%)	637 (34.00%)		
Occasionally	4870 (24.90%)	350 (18.70%)		
Never	8940 (45.70%)	889 (47.40%)		
Sleep duration			48.73	<0.001
<7	6923 (35.40%)	816 (43.50%)		
7–8	10549 (53.90%)	887 (47.30%)		
>9	2087 (10.70%)	173 (9.20%)		
Diabetes	1156 (5.90%)	191 (10.20%)	53.02	<0.001
Hyperlipidemia	358 (1.80%)	79 (4.20%)	48.58	<0.001
Hypertension	3122 (16.00%)	486 (25.90%)	120.92	<0.001

^a^ “Other” indicates that the subjects did not provide an answer.

**Table 2:** Prevalence odds ratio and 95% confidence intervals for correlates of gallbladder diseases to no gallbladder diseases (multivariate logistic regression).

Variable	Gallbladder Diseases (n = 1876)		Model 1	Model 2
	n	%	POR (95% CIs)	POR (95% CIs)
Residence (Rural)	1173	62.50	1.91 (1.74–2.11)	1.65 (1.49–1.82)
Female	1417	75.50	3.15 (2.83–3.51)	3.13 (2.76–3.55)
Age				
18–29	49	2.60	1.00	1.00
30–44	416	22.20	3.43 (2.54–4.62)	2.79 (2.06–3.77)
45–59	902	48.10	6.16 (4.60–8.24)	4.26 (3.17–5.73)
60–79	509	27.10	7.10 (5.28–9.57)	4.72 (3.48–6.41)
Smoking habits				
Never-smoker	1254	66.80	1.00	1.00
Current smoker	464	24.70	0.69 (0.62–0.78)	1.15 (1.01–1.31)
Former smoker	158	8.04	0.95 (0.80–1.13)	1.37 (1.13–1.65)
Seafood				
Few or never eat	1078	57.50	1.00	1.00
Eat everyday	134	7.10	0.52 (0.43–0.62)	0.77 (0.63–0.93)
Sometimes eat	664	35.40	0.64 (0.58–0.71)	0.84 (0.76–0.94)
Soy products				
Few or never eat	332	17.70	1.00	1.00
Eat everyday	1002	53.40	0.42 (0.36–0.48)	0.50 (0.44–0.58)
Sometimes eat	542	28.90	0.46 (0.40–0.53)	0.56 (0.48–0.65)
